# Treatment of the Hoffa fracture of the lateral femoral condyle with cannulated screws in combination with an anti-gliding steel plate, a retrospective observational study

**DOI:** 10.1186/s12893-022-01803-9

**Published:** 2022-10-03

**Authors:** Chunfu Huang, Zuchang Fu, Qingkeng Wang, Ruijin You, Feng Zhao

**Affiliations:** Department of Orthopaedics, No. 910 Hospital of PLA, Quanzhou, 362000 Fujian China

**Keywords:** Femoral fracture, Internal fixation, Bone screw, Bony plate

## Abstract

**Background:**

The stability of the Hoffa fracture fixed with a cannulated screw is limited. In the present work, we adopted two cannulated screws from anterior to posterior and posteriorly anti-gliding plate to treat 12 cases with Hoffa fracture of the lateral femoral condyle.

**Methods:**

Open reduction and internal fixation were performed in 12 patients with Hoffa fractures of the lateral femoral condyle. The Hoffa fracture end was fixed with an anti-gliding steel plate based on cannulated screw fixation in the rear of the distal femur. All patients were followed for 12–24 months, with a median of 15.3 ± 4.6 months.

**Results:**

All fractures were bony union, with a healing time of 3–6 months (median, 4.5 months). The Letenneur evaluation system was used to assess efficacy. Four indexes were observed, including knee range of motion (ROM), stability, pain, and dependent ambulation. The results revealed that eight cases were excellent and four cases good, with a good rate of 100%.

**Conclusion:**

The surgical treatment with cannulated screws in combination with an anti-gliding steel plate could fix the end of the Hoffa fracture, which could acquire strong internal fixation and a good curative effect.

## Introduction

The relatively rare Hoffa fracture refers to the coronal fracture of the distal medial and lateral condyle of the femur [1–5], accounting for 8.7 13% [5] of distal femoral fractures. High-energy injuries, such as road traffic injuries and high fall injuries, are the predominant causes of this fracture [6]. Hence, Hoffa fracture often occurs with injury to the distal end and adjacent part of the femur. The incidence rate of Hoffa fracture is relatively low, and its diagnosis is difficult. Nonoperative treatment can lead to fracture displacement and limited knee joint function. Currently, two or more cannulated screws are often used for anterior or posterior fixation in clinics. Cannulated screw fixation is considered to have the advantages of minor trauma, fewer complications, and reliable fixation [4, 5]. However, the stability of Hoffa fracture fixed with cannulated screw is limited, and there have been clinical reports of fixation failure. In the present work, we adopted two cannulated screws from anterior to posterior and posteriorly anti-gliding plate to treat 12 cases with Hoffa fracture of the lateral femoral condyle enrolled in our hospital from June 2015 to December 2019. The procedure obtained satisfactory results.

## Patients and methods

### Study design

Retrospective observational study.

### General data

A retrospective analysis of 12 cases with Hoffa fractures of the lateral femoral condyle admitted to our hospital from June 2015 to December 2019 was performed.

The following are the *inclusion criteria*:

- The patients had a history of trauma that was confirmed by X-ray, CT, and MRI before treatment;
- Patients had complete clinical data and were followed for at least 1 year.

Exclusion criteria

- Patients diagnosed with bicondylar Hoffa fracture;
- Patients with osteoarthritis of the knee joint before treatment;
- Patients who could not be treated surgically due to age, physical condition, etc.

### Surgical procedures

**Fig. 1:**
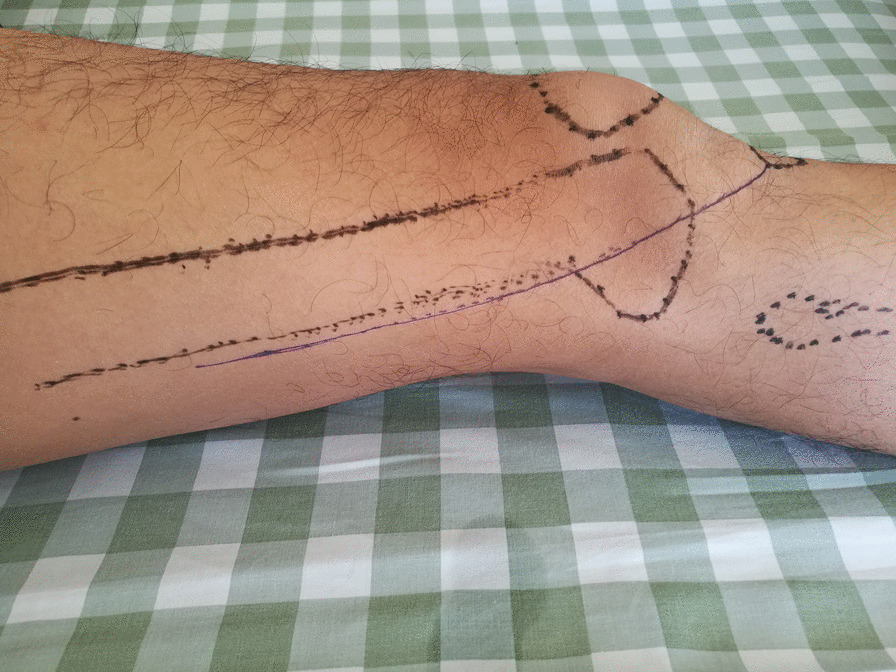
Schematic diagram of operative incision

**Fig. 2:**
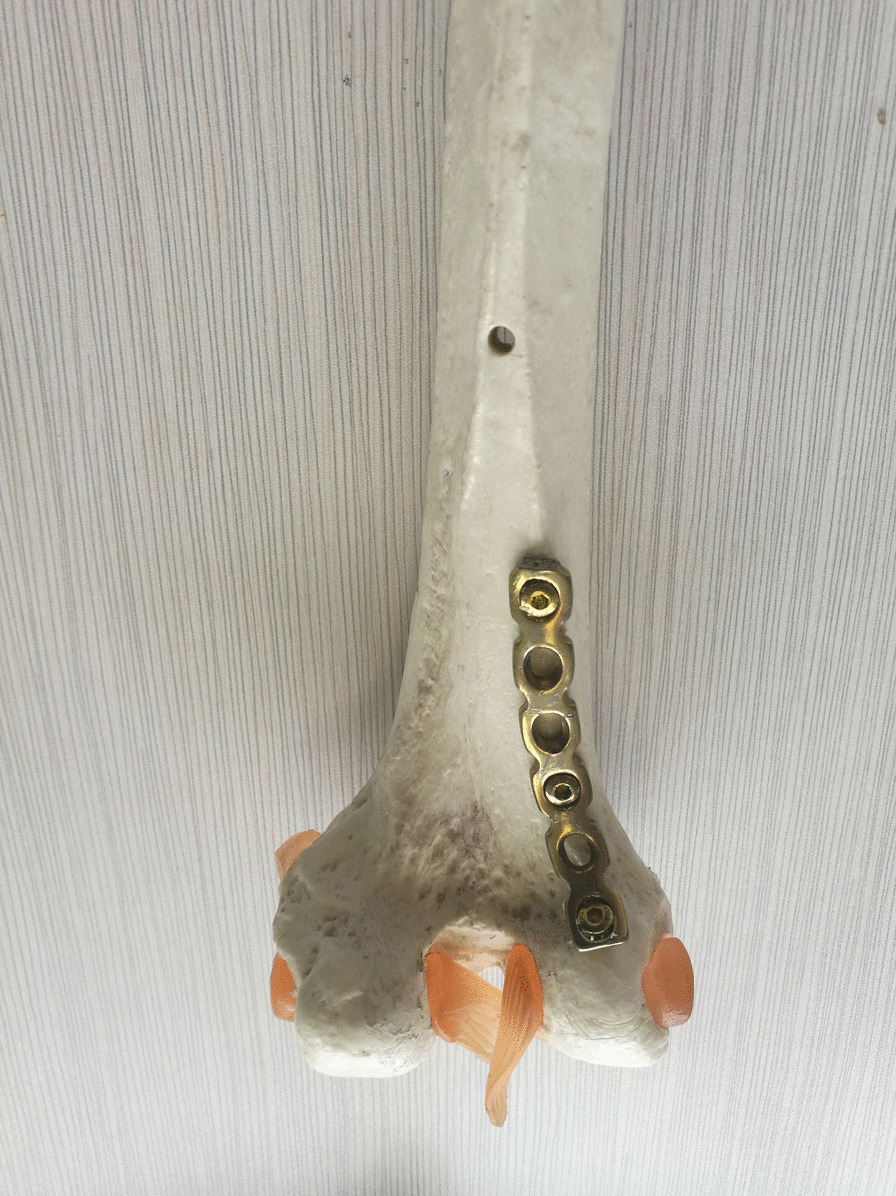
Schematic diagram of posterior anti-gliding plate fixation of lateral femoral condyle

**Fig. 3:**
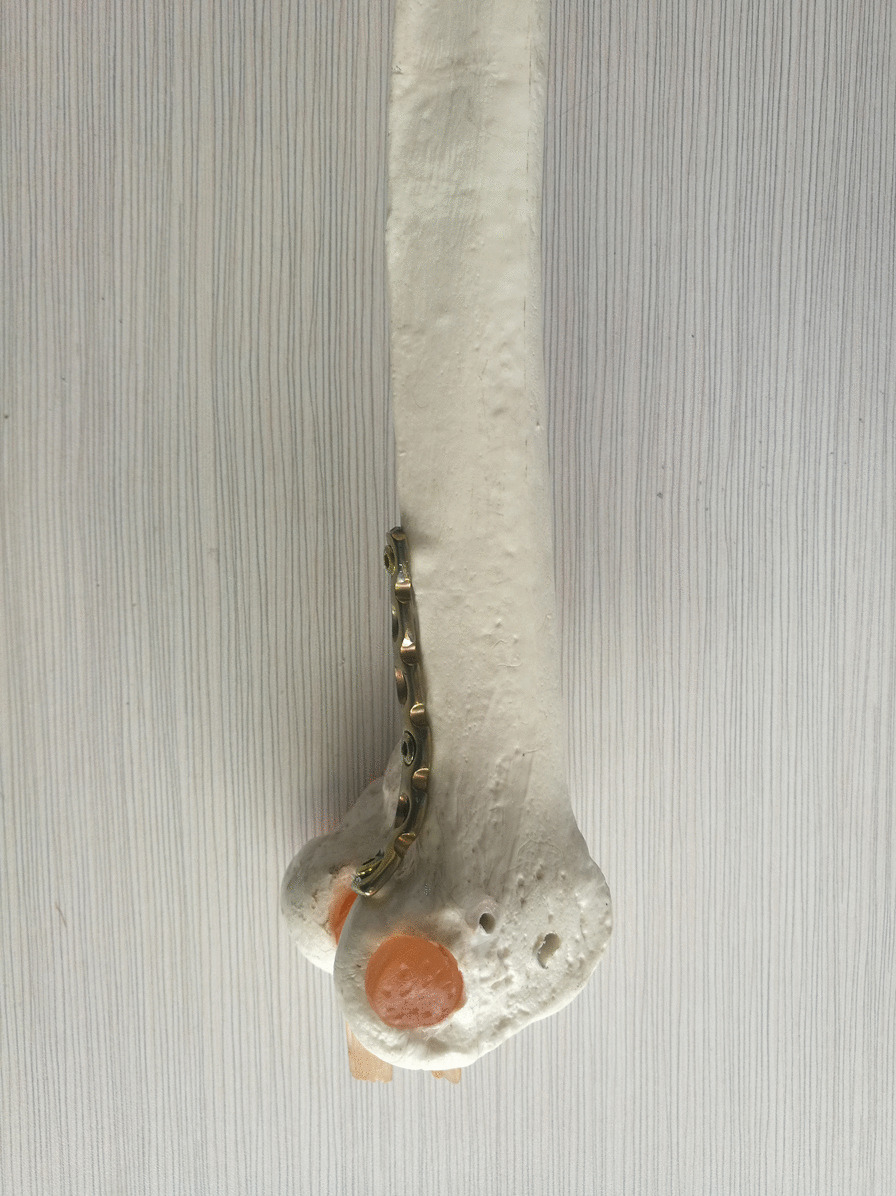
Schematic diagram of posterior anti-gliding plate fixation of lateral femoral condyle

All patients underwent open reduction and internal fixation 5–7 days after injury under combined spinal-epidural anesthesia. A healthy lateral position was taken to make a posterolateral femoral incision that extends from the lateral condyle of the femur to the tibial tubercle (Fig. 1). The articular capsule was cut open to expose the knee articular surface and the Hoffa fracture end, with a focus on not damaging the lateral knee collateral ligament and popliteal tendon. Intra-articular hemorrhage was cleared with knee flexion of 30° to restore the end of the Hoffa fracture under direct vision to ensure a smooth cartilage surface. Two 4.0 mm cannulated lag screws were drilled in the anteroposterior direction, perpendicular to the fracture line as much as possible; screw heads were buried below the cartilage of the articular surface, and the tails could not reach out of the articular surface of the contralateral posterior condyle. The fibrocartilaginous structure of the meniscus was probed, and the injured surface was shaped or repaired. The top of the partial plantar muscle and the lateral head of the gastrocnemius muscle were peeled off the posterior side of the lateral femoral condyle and on the surface of the posterior articular capsule; the width of the peel was about 1 cm, without cutting the posterior articular capsule open. The reconstruction plate was prebent and fixed on the posterior side of the lateral femoral condyle (Figs. 2 and 3) as an anti-gliding plate. The distal end of the plate failed to reach the articular surface of the posterior condyle of the femur, where a screw was drilled to fix the Hoffa fracture fragment. On the proximal, two screws were used to fix the reconstruction plate to resist the longitudinal shear force of the Hoffa fracture fragment. The stability of the internal fixation was evaluated by completely moving the knee joint prior to incision closure, as well as by stress test and T drawer test on the left side of the knee joint to determine its stability.

### Postoperative rehabilitation

External plaster fixation was not used in all patients postoperatively, and the drainage tube was removed when the postoperative drainage volume was < 20 mL/day. On the first day after the operation, an active knee flexion and extension exercise and passive knee joint function exercise with a rehabilitation device, continuous passive motion (CPM) for lower limb joints, were performed. The knee joint was moved fully and passively 1 to 2 times when dressing. The affected limb fell to the ground with free-weight walking aids 4 weeks later, and partial load walking started after 6 weeks. Full load walking was dependent on the growth of the callus after 12 weeks.

### Efficacy evaluations

The role of the knee joint was evaluated using the Letenneur standard [2]. Taking into account the range of movement, stability, pain, and assisted walking of the knee joint, the results were divided into four categories.

- *Excellent* Movement range > 120°, stable, no pain, no assisted walking;
- *Good* Movement range > 120°, stable, slightly pained during exercise, no assisted walking;
- *General* Movement range 90°–120°, stable and occasional pain after exercise, without assisted walking;
- *Poor* Movement range < 90°, unstable and frequently painful, assisted walking.

### Ethics approval and consent to participate

All methods were carried out according to relevant guidelines and regulations, and the China No. 910 Hospital of PLA Ethics Committee/Institutional Review Board has confirmed that our study was waived. Informed consent was obtained from all subjects and/or their legal guardians.

## Results

### Patient characteristics

In this single-center retrospective observational study, we evaluated the efficiency of treatment of two cannulated screws from anterior to posterior and posterior anti-gliding plate in 12 patients with Hoffa fractures of the lateral femoral condyle. According to the above criteria, in this study 12 cases were considered, including eight men and four women, 28 to 56 years of age (average age, 5.2 ± 9.9 years). Ten patients had sustained a road traffic injury, and two patients had a high falling injury. All fractures were closed, including four cases of meniscus injury. X-ray, CT, 3D reconstruction, and magnetic resonance examination were performed for all patients. There were 10 cases of the lateral condyle, type I, and two cases of type III fracture, according to the Letenneur system.

### Therapeutic efficacy in patients with Hoffa fracture of the lateral femoral condyle

This group of patients was followed for 12 to 24 months (average duration, 15.3 ± 4.6 months). All fractures healed in 3 to 6 months, with an average healing time of 4.5 months. No complications developed such as infection, fracture non-union, malunion, loosening of the internal fixation, fracture, necrosis of the femoral condyle, etc.

**Fig. 4:**
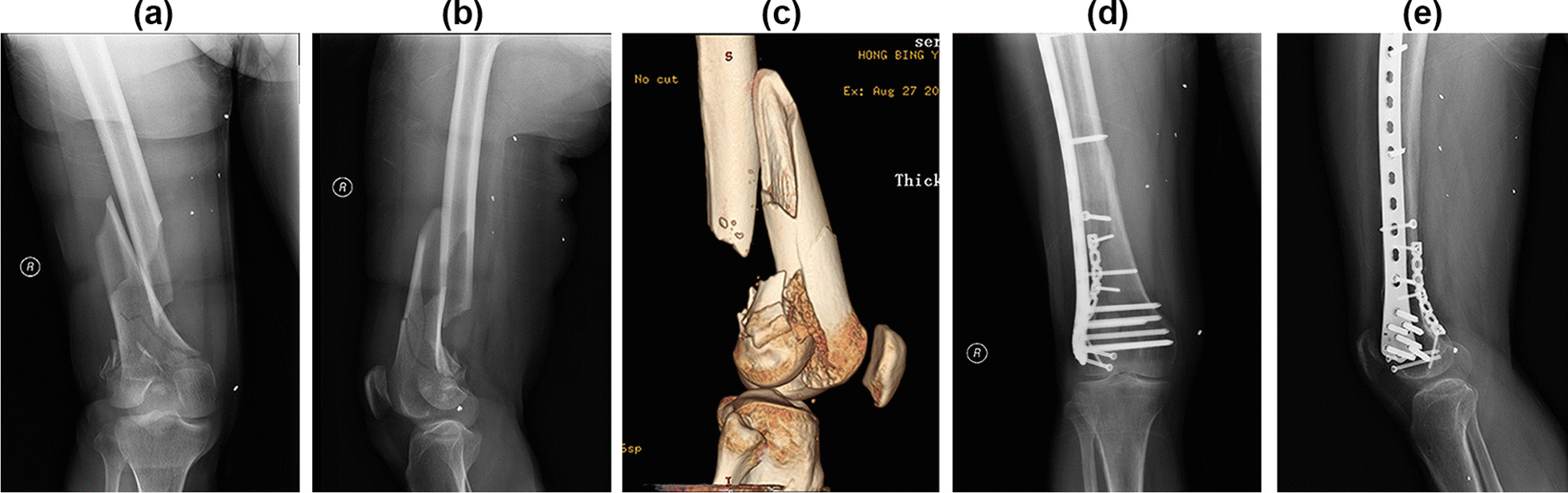
A 52-year-old man was injured on a motorcycle. **a**, **b** Preoperative anterior and lateral X-rays showed a fracture of the lower segment of the right femur and fracture of the right femoral condyle; **c** The three-dimensional CT showed a fracture of the lower right femur and coronal shear fracture of the right lateral femoral condyle; **d**, **e** Positive and lateral X-ray films showed fracture healing 3 months after the operation

In terms of knee joint function, eight cases were rated excellent, four cases good, and nil cases in the general and poor categories. The excellent and good cases were 60% and 40%, respectively. Figure 4 shows typical cases.

## Discussion

The Hoffa fracture refers to the coronal shear fracture of the medial or lateral condyle of the distal femur, which corresponds to type 33-B3 in the muller AO (Arbeitsgemeinschaft für Osteosynthesefragen) classification. The incidence of Hoffa fractures has increased with the rapid development of transportation, construction, and industry. This study investigates the therapeutic outcome of Hoffa fracture after cannulated screws in combination with an anti-gliding steel plate at a mean follow-up of 12–24 months after the operation. We used two 4.0 mm cannulated screws to fix the Hoffa fracture end vertically, and an anti-gliding plate was used behind the femoral condyle to resist the shear stress of the fracture end for firm fixation and early functional exercise. The outcome showed that the patients have an average healing time of 4.5 months and full functional recovery of the knee was presented. These findings enriched our understanding of the treatment of the Hoffa fracture of the lateral femoral condyle and suggested that cannulated screws might be useful against the Hoffa fracture of the lateral femoral condyle supplemented by an anti-gliding steel plate.

The Hoffa fracture with the involvement of the weight-bearing articular surface is an unstable intra-articular fracture. There is a large shear force at the end of the fracture, and the gastrocnemius muscle is often attached to the back of the end of the fracture. Therefore, the end of the fracture is extremely unstable and is prone to easy displacement, while prolonged braking and external fixation can cause knee joint stiffness. Therefore, early surgical treatment is recommended for Hoffa fractures, regardless of the displacement site [3]. Precise anatomical reposition and stable fixation are profoundly important to achieve good outcomes. The Hoffa fracture frequently occurs with damage to the distal and adjacent parts of the femur. The injury mechanism is that the knee joint is flexed and the axial force of the lower extremity exerts a significant shear force on the tibial plateau and femoral condyle, leading to a coronal shear fracture of the posterior femoral condyle [5, 6]. The axial force is exerted first on the lateral condyle of the femur due to the approximately 10° eversion of the dissected upper knee joint; therefore, the lateral Hoffa fracture is significantly greater than the medial one [7]. Clinically, Hoffa fractures of the lateral femoral condyle are commonly fixed with cannulated screws, but failures of simply using cannulated screws to fix Hoffa fractures are often reported due to inadequate mechanical strength [8]. The main reason is that this inadequate mechanical strength does not resist the shear stress of the fractured end during functional exercise. In our present work, we made a large incision to fully expose the end of the fracture and the lower femoral fracture and used steel plates and screws for firm internal fixation. An anti-gliding steel plate was applied to restore normal tibial and patellofemoral joints, facilitate early functional exercise, and prevent complications such as poor axial alignment, traumatic arthritis, and stiff and unstable knee joint therapy mitigating the incidence of fracture non-union and malunion. According to the evaluation of the role of the knee joint by the Letenneur standard [2], we found that eight cases were rated as excellent, four as good and nil cases in the general and poor categories. The excellent and good cases were 60% and 40%, respectively, suggesting that the combination with an anti-gliding steel plate reduces the risk of fracture block displacement.

## Conclusions

On the basis of the cannulated screw fixation, the author used an anti-gliding plate behind the femoral condyle to fix the fracture end, and a satisfactory clinical effect was obtained, which can be replicated. Therefore, cannulated screws should be combined with an anti-gliding steel plate in the treatment of Hoffa fracture of the lateral femoral condyl, providing a reference for clinical application. However, we did not obtain patient-reported outcome measures (PROM) for each patient and just offered surgeon-related perspectives. The reason may be related to the fact that we just evaluated the efficiency of treatment by radiographs, CT, 3D reconstruction, and MRI in patients. Most importantly, we did not compare the treatment efficiency of cannulated screws in combination with an anti-gliding steel plate or not due to the limited number of patients and limited patient material.

## Data Availability

The datasets used and/or analyzed during the current study are available from the corresponding author on reasonable request.

## Abbreviations

ROM: Range of motion
CPM: Continuous passive motion
AO: Arbeitsgemeinschaft für Osteosynthesefragen
